# Synthesis, Crystal Structures, and DNA Binding Properties of Zinc(II) Complexes with 3-Pyridine Aldoxime

**DOI:** 10.1155/2010/803424

**Published:** 2010-11-02

**Authors:** Konstantis F. Konidaris, Rigini Papi, Eugenia Katsoulakou, Catherine P. Raptopoulou, Dimitrios A. Kyriakidis, Evy Manessi-Zoupa

**Affiliations:** ^1^Department of Chemistry, University of Patras, 265 04 Patras, Greece; ^2^Department of Chemistry, Aristotle University of Thessaloniki, 541 24 Thessaloniki, Greece; ^3^Institute of Materials Science, NCSR “Demokritos”, 153 10 Aghia Paraskevi Attikis, Greece

## Abstract

The employment of 3-pyridine aldoxime, (3-py)CHNOH, in Zn^II^ chemistry has afforded two novel compounds: [Zn(acac)_2_{(3-py)CHNOH}]·H_2_O (**1**·H_2_O) [where acac^−^ is the pentane-2,4-dionato(-1) ion] and [Zn_2_(O_2_CMe)_4_{(3-py)CHNOH}_2_] (**2**). Complex **1**·H_2_O crystallizes in the monoclinic space group *P*2_1_/*n*. The Zn^II^ ion is five-coordinated, surrounded by four oxygen atoms of two acac^−^ moieties and by the pyridyl nitrogen atom of the (3-py)CHNOH ligand. Molecules of **1** interact with the water lattice molecules forming a 2D hydrogen-bonding network. Complex **2** crystallizes in the triclinic *P*-1 space group and displays a dinuclear paddle-wheel structure. Each Zn^II^ exhibits a perfect square pyramidal geometry, with four carboxylate oxygen atoms at the basal plane and the pyridyl nitrogen of one monodentate (3-py)CHNOH ligand at the apex. DNA mobility shift assays were performed for the determination of the *in vitro* effect of both complexes on the integrity and the electrophoretic mobility of pDNA.

## 1. Introduction

During the last decades, there has been considerable interest in the interaction of small molecules with DNA [1, 2]. DNA is generally the primary intracellular target of anticancer drugs, so such interactions can cause damage in cancer cells, block their division and consequently result in cell death [3]. Small synthetic binders can interact with DNA through the following three noncovalent modes: intercalation, groove binding, and external static electronic effects [4]. Transition metal complexes are a particularly interesting class of DNA-binders because of their cationic character, well-defined three-dimensional structure, aptitude to perform hydrolysis and redox reactions, as well as extensively developed substitution chemistry that allows easy modulations of their binding and reactive properties [5]. 

Among the various metal ions studied with nucleic acids and nucleobases, Zn^II^ has occupied a special position [6], mainly due to the following reasons [7]: Zn^II^ is a strong Lewis acid and exchanges ligands very rapidly; is of low toxicity; it has no redox chemistry, catalyzing only hydrolytic cleavage of DNA. For all the above mentioned reasons, the binding of Zn^II^ complexes with DNA has attracted much attention [8, 9]. It has been reported that the binding properties of the complexes depend on several factors, such as the coordination geometry, the type of the donor-atoms and the planarity of ligands [10]. 

**Scheme 1:**
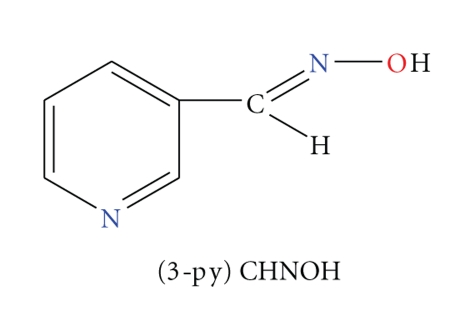
Structural formula and abbreviation of the ligand employed in this work.

The ligand used in this work (Scheme 1) belongs to the family of pyridyl oximes. The coordination chemistry of these compounds has been extensively explored over the last two decades, mainly with paramagnetic 3d metal ions towards new molecular materials with interesting magnetic properties [11]. As a consequence, the diamagnetic character of the Zn^II^ ion has led to a “gap” in the literature, concerning the area of the coordination chemistry of oximes. Recently, we have tried to fill this gap by the use of simple pyridyl oximes (the term “simple” means here ligands with only one pyridyl and one oxime group as donors) in Zn^II^ coordination chemistry. We reported the largest up to date Zn(II)/oxime cluster [12], as well as the first complexes of Zn^II^ with 3- and 4-pyridine aldoxime [13]. 

In this study, our efforts were initiated by the synthesis and characterization of new Zn^II^/3-pyridine aldoxime complexes, while our next objective was to investigate the interaction of these compounds with plasmid DNA. The structural formula of the free ligand is illustrated in Scheme 1.

## 2. Experiments

### 2.1. Starting Materials and Physical Measurements

All manipulations were performed under aerobic conditions using reagents and solvents as received. Zinc acetylacetonate, zinc acetate, and 3-pyridinealdoxime were purchased from Aldrich Co. Elemental analyses (C, H, N) were performed by the University of Ioannina (Greece) Microanalytical Laboratory using an EA 1108 Carlo Erba analyzer. IR spectra (4000–450 cm^−1^) were recorded on a Perkin-Elmer 16 PC FT-IR spectrometer with samples prepared as KBr pellets. 

pDNA isolation was performed from a fully grown culture of *Escherichia coli *Top10F^−^ harboring the pBluescript plasmid. The Macherey-Nagel plasmid DNA isolation kit was used. All plastics and glassware used in the experiments were autoclaved for 30 min at 120°C and 130 Kpa.

### 2.2. *Compound Preparation*

#### 2.2.1. Preparation of [Zn(acac)_2_{(3-py)CHNOH)}]·H_2_O (**1**·H_2_O)

Zn(acac)_2_·H_2_O (0.210 g, 0.80 mmol) was suspended in MeOH (10 cm^3^) and then dissolved upon stirring by adding a solution of (3-py)CHNOH (0.195 g, 1.60 mmol) in the same solvent (10 cm^3^). The resulting colourless solution was stirred at ambient temperature for 30 min and allowed to slowly evaporate at room temperature. Well-formed, X-ray quality colourless crystals of the product appeared within a period of four days. The crystals were collected by vacuum filtration, washed with cold MeOH (2 × 2 cm^3^) and Et_2_O (3 × 5 cm^3^), and dried in air. The yield was ca. 65%. Found %: C, 47.60; H, 5.84; N, 6.73. Calc % for C_16_H_24_N_2_O_6_Zn: C, 47.36; H, 5.96; N, 6.90. IR data (KBr, cm^−1^): 3468 (wb), 3196 (wb), 3084 (wb), 2998 (w), 2960 (w), 2914 (w), 2804 (w), 1956 (vw), 1654 (sh), 1586 (s), 1552 (vs), 1400 (s), 1340 (sh), 1316 (sh), 1268 (m), 1190 (m), 1124 (w), 1102 (vw), 1058 (vw), 1020 (w), 986 (m), 928 (w), 890 (vw), 818 (vw), 770 (w), 702 (w), 654 (w), 560 (w), 528 (vw), and 464 (vw).

#### 2.2.2. Preparation of [Zn_2_(O_2_CMe)_4_{(3-py)CHNOH}_2_] (**2**)

Zn(O_2_CMe)_2_·2H_2_O (0.110 g, 0.50 mmol) was suspended in Me_2_CO (10 cm^3^) and then dissolved upon stirring by adding a solution of (3-py)CHNOH (0.122 g, 1.00 mmol) in the same solvent (10 cm^3^). The resulting colourless solution was stirred at ambient temperature for 30 min and then layered with n-hexane (40 cm^3^). Slow mixing gave well-formed, X-ray quality crystals of the product. The colourless crystals were collected by filtration, washed with cold Me_2_CO (2 × 3 cm^3^) and Et_2_O (2 × 3 cm^3^), and dried in air. The yield was ca. 53%. Found %: C, 39.10; H, 4.01; N, 9.23. Calc % for C_20_H_24_N_4_O_10_Zn_2_: C, 39.34; H, 3.94; N, 9.17. IR data (KBr, cm^−1^): 3454 (wb), 3192 (m), 3084 (m), 2996 (m), 2962 (m), 2914 (m), 2808 (w), 1656 (sh), 1586 (vs), 1522 (s), 1400 (vs), 1340 (s), 1316 (s), 1268 (s), 1192 (w), 1124 (w), 1102 (vw), 1058 (vw), 1020 (m), 986 (s), 928 (m), 890 (w), 818 (m), 770 (m), 702 (m), 682 (w) 654 (w), 562 (w), and 418 (vw).

### 2.3. X-Ray Crystal Structure Determination

Crystals of **1**·H_2_O (0.22 × 0.34 × 0.45 mm) and **2** (0.12 × 0.14 × 0.24 mm) were mounted in capillary. Diffraction measurements for **1**·H_2_O were made on a Crystal Logic Dual Goniometer diffractometer using graphite monochromated Mo radiation, and for **2 **on a P2_1_ Nicolet diffractometer upgraded by Crystal Logic using graphite monochromated Cu radiation. Unit cell dimensions were determined and refined by using the angular settings of 25 automatically centered reflections in the ranges of 11 < 2*θ* < 23° (for **1**·H_2_O) and 22 < 2*θ* < 54° (for **2**) and they appear in Table 1. Intensity data were recorded using a *θ*-2*θ* scan. Three standard reflections monitored every 97 reflections showed less than 3% variation and no decay. Lorentz, polarization and psi-scan absorption (only for **1**·H_2_O) corrections were applied using Crystal Logic software. The structures were solved by direct methods using SHELXS-97 [14] and refined by full-matrix least-squares techniques on F^2^ with SHELXL-97 [15]. Further experimental crystallographic details for **1**·H_2_O: 2*θ*_max_ = 50°; reflections collected/unique/used, 3449/3284 [R_int _ = 0.0179]/3284; 269 parameters refined; (Δ/*σ*)_max_ = 0.001; (Δ*ρ*)_max _/(Δ*ρ*)_min_ = 0.453/−0.457 e/Å^3^; *R*1/w*R*2 (for all data), 0.0371/0.834. Further experimental crystallographic details for **2**: 2*θ*max  = 118°; reflections collected/unique/used, 1964/1820 [R_int _ = 0.0538]/1820; 167 parameters refined; (Δ/*σ*)_max_ = 0.000; (Δ*ρ*)_max _/(Δ*ρ*)_min_ = 0.559/−0.553 *e*/Å^3^; *R*1/w*R*2 (for all data), 0.0524/0.1205. Hydrogen atoms were either located by difference maps and were refined isotropically or were introduced at calculated positions as riding on bonded atoms. All nonhydrogen atoms were refined anisotropically. CCDC codes 789660 and 789661 contain the supplementary crystallographic data for this paper. This data can be obtained free of charge at www.ccdc.cam.ac.uk/conts/retrieving.html [or from the Cambridge Crystallographic Data Centre, 12 Union Road, Cambridge CB2 1EZ, UK; Fax: ++44-1223-336 033; E-mail: deposit@ccdc.cam.ac.uk].

### 2.4. Effect on pDNA

0.1 *μ*g of pBluescript were incubated at 25°C in the presence of various concentrations of the complexes under study. After 1h at 25°C the reaction was terminated by the addition of loading buffer consisting of 0.25% bromophenol blue, 0.25% xylene cyanol FF and 30% (v/v) glycerol in water. The products resulting from DNA-compound interactions were separated by electrophoresis on agarose gels (1% w/v), which contained 1 *μ*g/ml ethidium bromide (EtBr) in 40 mM Tris–acetate, pH 7.5, 20 mM sodium acetate, 2 mM Na_2_EDTA, at 5 V/cm. Agarose gel electrophoresis was performed in a horizontal gel apparatus (Mini-Sub*™* DNA Cell, BioRad) for about 4 h. The gels were visualized in the presence of UV light. All assays were duplicated.

## 3. Results and Discussion

### 3.1. Synthetic Comments

Our previous investigation on the reaction between Zn(O_2_CPh)_2_ and (3-py)CHNOH led to a trinuclear benzoate cluster [13]. In a subsequent step, we expanded our research to similar or different types of bidentate ligands, such as MeCO_2_^−^ and acac^−^, respectively. 

Our initial efforts involved the reaction of Zn(acac)_2_·H_2_O with one equivalent of (3-py)CHNOH in MeOH, which afforded colourless parallelepiped crystals of **1**·H_2_O upon slow evaporation of the reaction solution. Its formation can be represented by the equation (1)

(1) $$\begin{array}{cc} & \text{Zn}{\left(\text{acac}\right)}_{2}\cdot {\text{H}}_{2}\text{O}+\left(3\text{-py}\right)\text{CHNOH}\\ & \;\overset{\text{MeOH}}{\to }\left[Z\text{n}{\left(\text{acac}\right)}_{2}\left\{\left(3\text{-py}\right)\text{CHNOH}\right\}\right]\cdot {\text{H}}_{2}\text{O}\;\\ & \;\;\;\;\;\;\;\;\;1\cdot {\text{H}}_{2}\text{O} \end{array}$$

Treatment of Zn(O_2_CMe)_2_·2H_2_O with two equivalents of (3-py)CHNOH in Me_2_CO afforded colourless parallelepiped crystals of **2** upon layering of the reaction solution with n-hexane. The chemically balanced equation for the synthesis of **2** is:

(2) $$\begin{array}{cc} & 2\;\text{Zn}{\left({\text{O}}_{2}\text{CMe}\right)}_{2}\cdot 2{\text{H}}_{2}\text{O}+2\left(3\text{-py}\right)\text{CHNOH}\\ & \;\overset{{\text{Me}}_{2}\text{CO}}{\to }\left[Z{\text{n}}_{2}{\left({\text{O}}_{2}\text{CMe}\right)}_{4}{\left\{\left(3\text{-py}\right)\text{CHNOH}\right\}}_{2}\right]+4{\text{H}}_{2}\text{O}\\ & \;\;\;\;\;\;\;\;\;\;\;2 \end{array}$$

As a next step, we tried to modify the structural identity of **1**·H_2_O by using excess of Zn(acac)_2_·H_2_O. A probable result would be the isolation of a paddle wheel structure with four bidentate bridging acac^−^ ligands and two monodentate (3-py)CHNOH ligands, that is, a structure analogous to that of compound **2**. Unfortunately, our efforts did not yield fruits; all the reactions lead to the isolation of solids corresponding to compound **1**·H_2_O, emphasizing the reduced (compared to carboxylates) bridging capability of acac^−^.

### 3.2. Description of Structures

**Figure 1:**
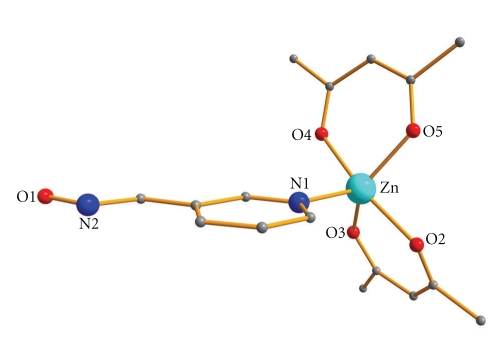
Partially labeled plot of the molecular structure of **1**·H_2_O. H atoms and the solvate H_2_O molecule have been omitted for clarity.

**Figure 2:**
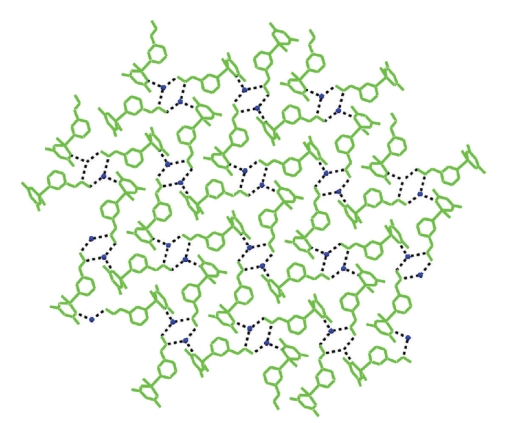
A part of the 2D network in the crystal lattice of **1**·H_2_O due to hydrogen bonding interactions (dashed lines). Oxygen atoms of the water lattice molecules are represented by blue spheres.

**Figure 3:**
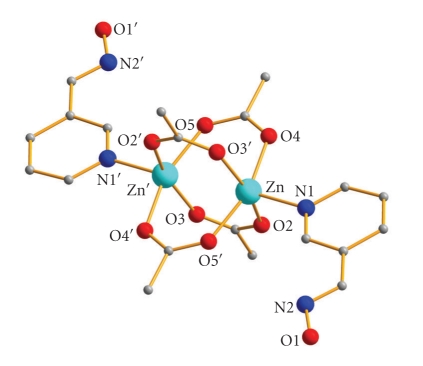
Partially labeled plot of the molecular structure of **2**· H atoms have been omitted for clarity.

**Figure 4:**
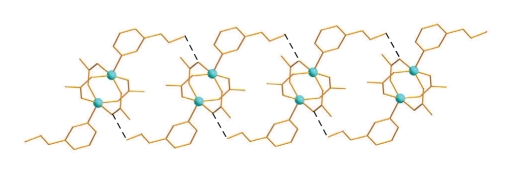
A small portion of the ladder-like 1D architectures of **2** due to H-bonding interactions (black dashed lines). The H atoms have been omitted for clarity.

Aspects of the molecular and crystal structures of complexes **1**·H_2_O and **2** are shown in Figures 1–4. Selected interatomic distances and angles are listed in Tables 2 and 3, while important hydrogen bonding interactions are presented in Table 4.

Complex **1**·H_2_O crystallizes in the monoclinic space group *P*2_1_/*n*. Its crystal structure consists of mononuclear [Zn(acac)_2_{(3-py)CHNOH}] molecules and H_2_O molecules being present in the lattice. The metal center is five-coordinated surrounded by two acetylacetonate (acac^−^) and one (3-py)CHNOH ligand. Each of the acac^−^ moiety acts in a bidentate chelating way, while the (3-py)CHNOH behaves as a monodentate ligand via the nitrogen atom of the pyridine ring. The coordination geometry of the Zn^II^ ion is heavily distorted and thus it can be either described as distorted square pyramidal or as distorted trigonal bipyramidal. Analysis of the shape-determining angles using the approach of Reedijk and coworkers [16] yields a trigonality index, *τ*, value of 0.53 (*τ* = 0 and 1 for perfect sp and tbp geometries, respectively). By adopting the square pyramidal geometry, the basal plane is occupied by four acetylacetonate oxygen atoms, while the apical position is taken by the pyridyl nitrogen atom of the oxime ligand. Adopting the trigonal bipyramidal description, the axial positions are occupied by O(2) and O(4) and the equatorial ones by O(3), O(5), and N(1). 

In the crystal lattice of **1**·H_2_O, the molecules of **1** interact with the water lattice molecules through hydrogen bonds, forming a 2D network (Figure 2, Table 4). 

Complex **2** is a new member of Zn(II) carboxylate complexes with a paddle wheel structure [17–20]. The Zn^II^ ions are bridged by four *s* *y* *n*, *s* *y* *n*-*η* ^1^:*η* ^1^:*μ* MeCO_2_^−^ ligands and each one has a perfect square pyramidal coordination geometry (*τ* = 0.01), with the apex provided by the pyridyl nitrogen atom of a monodentate (3-py)CHNOH ligand. The Zn⋯Zn distance is 2.923(2)Å, while each Zn^II^ ion lies 0.386 Å out of its least-squares basal plane towards the pyridyl nitrogen atom. The mean Zn–O(carboxylate) bond length is approximately 2.044 Å which is typical and unremarkable [21]. There is a crystallographically imposed inversion center in the midpoint of the Zn⋯Zn distance.

In the crystal lattice of **2**, the dinuclear molecules interact through hydrogen bonds. Both oxime groups act as donors to carboxylate oxygen atoms, forming double, ladder-like chains along the c axis (Table 4, Figure 4).

### 3.3. IR Spectroscopy

The IR spectra of **1**·H_2_O and **2 **exhibit weak bands at 3468 and 3454 cm^−1^, respectively, assignable to the *ν*(OH) vibration of the coordinated pyridyl oxime ligands [22]. The broadness and relatively low frequency of these bands are both indicative of hydrogen bonding. The medium intensity bands at 1636 and 1124 cm^−1^ in the spectrum of the free ligand (3-py)CHNOH are assigned to *ν*(C=N)oxime and *ν* (N–O)oxime, respectively [23]. In the spectra of the complexes, these bands are observed at approximately the same wavenumbers, confirming the nonparticipation of the oxime group in coordination. The in-plane deformation band of the pyridyl ring of free (3-py)CHNOH (638 cm^−1^) shifts upwards (654 cm^−1^) in the spectra of **1**·H_2_O and **2, **confirming the crystallography established involvement of the ring-N atom in coordination [24]. 

The presence of chelating acac^−^ ligands in complex **1**·H_2_O is reflected by the presence of two strong intensity bands at 1500–1600 cm^−1^. The higher frequency band (1586 cm^−1^) is attributed to *ν*(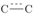)coupled with *ν*(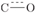), while the lower frequency band (1552 cm^−1^) is attributed to *ν*(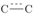)coupled with *ν*(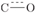) [25]. 

The strong intensity bands at 1522 and 1400 cm^−1^ in the spectrum of **2** are assigned to the *ν*_as_(CO_2_) and *ν*_*s*_(CO_2_) modes of the acetate ligands, respectively [26]; the former may also involve a pyridyl stretching character. The difference Δ, where Δ = *ν*_as_(CO_2_) − *ν*_s_(CO_2_), is 122 cm^−1^, less than that for NaO_2_CMe (164 cm^−1^), as expected for bidentate bridging ligation [26].

### 3.4. Effect on pDNA

DNA mobility shift assays were carried out to investigate the ability of complexes **1**·H_2_O and **2**, as well as that of the (3-py)CHNOH free ligand to interact with plasmid DNA. The initial amount of pDNA was incubated with increasing concentrations of the tested compounds. When circular pDNA is subjected to electrophoresis, relatively fast migration will be observed for the supercoiled form (form I). If scission occurs on one strand (nicking), the supercoils will relax to generate a slower-moving open relaxed form (form II) [27]. If both strands are cleaved, a linear form (form III) will be generated and migrate between forms I and II [28]. 

**Figure 5:**
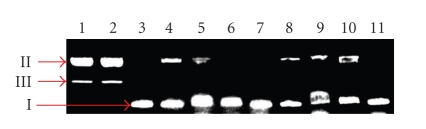
Agarose gel electrophoresis pattern of pDNA in the presence of the synthesized complexes and (3-py)CHNOH in various concentrations. Lane 1, DNA + **1**·H_2_O (5 mM); lane 2, DNA + **2 **(5 mM); lane 3, DNA+ (3-py)CHNOH (5 mM); lane 4, DNA + **1**·H_2_O (2.5 mM); lane 5, DNA + **2 **(2.5 mM); lane 6, DNA + (3-py)CHNOH (2.5 mM); lane 7, DNA + **1**·H_2_O (1 mM); lane 8, DNA + **2 **(1 mM); lane 9, DNA + **1**·H_2_O (0.1 mM); lane 10, DNA + **2 **(0.1 mM); lane 11, DNA control.

Figure 5 shows the gel electrophoretic separations of pDNA after incubation with **1**·H_2_O, **2 **and (3-py)CHNOH at various concentrations. Both complexes can break the double strand of pDNA and convert it to the relaxed form (II) and in a less extent to its linear form (III), at a concentration of 5 mM (Figure 5, lanes 1 and 2). At lower concentrations, the complexes display a minor effect on the integrity and electrophoretic mobility of pDNA, whereas the latter remains mostly in the supercoiled form (I). The (3-py)CHNOH ligand (Figure 5, lanes 3 and 6) does not display any interaction.

## 4. Conclusions

Two new complexes of Zn^II^, with 3-pyridine aldoxime as ligand, have been synthesized and characterized by single-crystal X-ray crystallography, elemental analyses, and IR spectroscopy. In both structures, (3-py)CHNOH acts as a monodentate ligand via the pyridyl nitrogen, while the oxime group does not participate in coordination. This coordination mode is the only one observed in complexes of (3-py)CHNOH with any metal up to date. Complexes **1**·H_2_O and **2 **are the second and the third structurally characterized Zn(II) complexes of (3-py)CHNOH.

The two complexes affect both the integrity and electrophoretic mobility of pDNA. At the highest tested concentration, **1**·H_2_O and **2 **are able to totally convert the supercoiled form of pDNA to the relaxed form and in less extent to its linear form. Other types of DNA-binding experiments are currently in progress in order to determine the way of interaction with pDNA. In the future, synthetic efforts with different types of anionic ligands (e.g., NO_3_^−^, SO_4_^2−^) can lead to a variety of (3-py)CHNOH complexes with potentially interesting DNA-binding properties.

## Figures and Tables

**Table 1:** Crystallographic data for complexes **1**·H_2_O and **2**.

Parameter	**1**·H_2_O	**2**
Formula	C_16_H_22_N_2_O_6_Zn	C_20_H_24_N_4_O_10_Zn_2_
Fw	403.73	611.17
Space group	*P*2_1_ */n *	*P*-1
*a* (Å)	10.531(4)	7.934(5)
*b* (Å)	15.779(5)	10.153(6)
*c* (Å)	11.602(4)	8.392(5)
*α* (°)	90	70.05(2)
*β* (°)	101.17(1)	87.34(2)
*γ* (°)	90	89.07(3)
*V* (Å^3^)	1891.4(11)	634.8(7)
Z	4	1
*T *(°C)	25	25
Radiation	Mo K*α*	Cu K*α*
*ρ*_calcd_ (g cm^−3^)	1.418	1.599
*μ* (mm^−1^)	1.331	2.856
Reflections with *I* > 2*σ*(*I*)	2865	1585
*R*_1_^*a*^	0.0302	0.0438
*w* *R*_2_^*a*^	0.0785	0.1125

^*a*^ *w* = 1/[*σ* ^2^(*F*_*o*_^2^) + (*α* *P*)^2^ + *b* *P*] and *P* = (max *F*_o_^2^, 0) + 2*F*_c_^2^)/3

*R*_1_ = Σ(|*F*_o_|-|*F*_o_|)/Σ(|*F*_o_|) and *w* *R*_2_ = {Σ[*w*(*F* *o* ^2^-*F*_*c*_^2^)^2^]/Σ[*w*(*F*_o_^2^)^2^]}^1/2^.

**Table 2:** Selected interatomic distances (Å) and angles (°) for complex **1**·H_2_O.

Zn-O(2)	2.055(2)	Zn-O(3)	1.988(2)
Zn-O(4)	2.029(2)	Zn-O(5)	2.000(2)
Zn-N(1)	2.073(2)		

O(3)-Zn-O(5)	137.1(9)	O(4)-Zn-O(2)	169.1(8)
O(3)-Zn-O(4)	88.5(8)	O(3)-Zn-N(1)	113.1(8)
O(5)-Zn-O(4)	89.6(7)	O(5)-Zn-N(1)	109.7(8)
O(3)-Zn-O(2)	89.00(8)	O(4)-Zn-N(1)	94.5(8)
O(5)-Zn-O(2)	85.1(7)	O(2)-Zn-N(1)	96.3(7)

**Table 3:** Selected interatomic distances (Å) and angles (°) for complex **2** ^*a*^.

Zn-O(2)	2.088(3)	Zn-O(4)	2.016(3)
Zn-O(3′)	2.055(3)	Zn-O(5′)	2.016(3)
Zn-N(1)	2.049(4)	Zn⋯Zn′	2.923(2)

O(4)-Zn-O(5′)	159.9(1)	N(1)-Zn-O(3′)	101.4(1)
O(4)-Zn-N(1)	100.4(1)	O(4)-Zn-O(2)	87.0(1)
O(5′)-Zn-N(1)	99.6(1)	O(5′)-Zn-O(2)	90.2(1)
O(4)-Zn-O(3′)	88.1(2)	N(1)-Zn-O(2)	98.4(1)
O(5′)-Zn-O(3′)	87.8(1)	O(3′)-Zn-O(2)	160.2(1)

^a^Symmetry transformations used to generate equivalent atoms: (′)-*x*, 1-*y*, −*z*.

**Table 4:** Hydrogen bonding interactions in **1**·H_2_O and **2**.

Interaction^a^D-H⋯A	D⋯A (Å)	H⋯A (Å)	D-H⋯A (°)	Symmetry operation of A
**1**·H_2_O				
O(1W)-H(1WA)⋯O(2)	3.002	2.5600	115.0	1/2 − *x*, −1/2 + *y*, 1/2 − *z*
O(1)-H(1O)⋯O(1W)	2.663	1.970	168.0	1 − *x*, −*y*, −*z*
O(1W)-H(1WB)⋯N(2)	3.002	2.300	145.0	*x*, *y*, *z*
**2**				
O(1)-H(1O)⋯O2	2.712	1.910	166.0	*x*, *y*, −1 + *z*

^a^A = acceptor, D = donor.
